# Virulence of the Seminal Fluid in Secondary Syphilis

**Published:** 1896-07

**Authors:** 


### VIRULENCE OF THE SEMINAL FLUID IN SECONDARY SYPHILIS.

Notwithstanding all the study which has been given to syphilis, facts present themselves from time to time which contradict the usual experience, and which are difficult of explanation. As regards certain points there has been, and is, much diversity of opinion. It is generally held that the physiological secretions of syphilitic subjects do not transmit the disease unless contaminated by mixture with products of syphilitic lesions. It is doubtful, however, whether this should be acknowledged as a universal rule. As having a bearing upon some of these debatable questions, we have read with interest the report of two remarkable cases by Dr. Rochon. Toward the close of 1893 this writer had occasion to observe a young married woman, 24 years of age, who presented a large chancre upon the abdominal wall, in the median line and about four finger-breadths below the umbilicus. The sore was nearly as large as a two-franc piece and had begun fifteen days previously upon the site of a slight abrasion of the skin, where it had been pinched by the corset. At the date of examination its aspect was typically that of a chancre.

The husband, 29 years of age, confessed that he had syphilis for two years. He had, on that account, delayed his marriage as long as he could without exciting family difficulties, and, at all events, until several months had elapsed without any syphilitic manifestation. Since marriage, although he had examined himself scrupulously and without cessation, he had never detected any sign which seemed to him in the slightest degree suspicious. He had been under careful treatment for eighteen months, but, on account of disordered stomach had avoided all medication for the preceding six months. Fearful of having a syphilitic child, he had been in the habit of ejaculating upon the abdomen of his wife, to whom he made the excuse that her health was too delicate to allow pregnancy.

Rigid inspection by Dr. Rochon failed to reveal any evidence of syphilis upon the skin or mucous membranes. Three weeks after she was first seen the woman exhibited a generalized papular eruption confirmatory of the diagnosis. The chancre was in process of cicatrization. At the end of a month the manifestations had disappeared and the writer lost sight of the patient.

The writer was unable to explain the case without admitting the inoculability of the semen, although he was able to find in medical literature only negative instances regarding that mode of contagion. Nevertheless, he remained in doubt until he encountered another case which seemed to throw light upon the subject.

In November, 1895, a young man, aged 26 years, syphilitic for fourteen months, and who also suffered from blenorrhagia, came under his care. No signs of syphilis were present at the time. The urethral discharge had ceased, except that pressure along the urethra from behind forward in the morning and before micturition forced out a drop of colorless, transparent and slightly viscid fluid. This fluid contained no gonococci. He was permitted to indulge in sexual congress. Five weeks later he returned, bringing with him his mistress, who complained of excoriations upon the vulva. The reporter was not a little surprised to find an indurated chancre of the fourchette without any trace of blenorrhagia. The woman most positively asserted that she had had relations with no other man for three months. The man had no sore upon the penis or within the mouth, but the slight urethral discharge continued. Both man and woman were subjected to specific treatment. In little more than a month the urethral discharge had much diminished and the chancre was in course of cure. In due time a disseminated papular rash came out upon the body and some mucous patches developed in the mouth. In this case the source of infection was conjectured by the author to be mucous patches in the urethra, excited by the gonorrhœal inflammation and persisting after the cure of the latter. The theory seemed to be supported by the disappearance of the discharge after the employment of specific remedies. No alteration of the epididymis, testicle or prostate was detected. This second case led the reporter to believe that the chancre of the abdomen had been produced in a similar manner by contamination of the semen through the ducts. This would be to admit the possibility of secondary syphilitic manifestations of the ejaculatory ducts. As at the present time we are aware that in this stage the liver, intestine, kidney, pleura, lung, etc., may be attacked, it does not seem difficult to suppose that the mucous membrane of the internal genital passages may likewise be involved. These parts are known to be frequently diseased during the tertiary period. Furthermore, a secondary epididymitis is described among what may be termed the classical manifestations of the malady. This lesion develops most often in the head of the epididymis, rarely in the tail. The tumor is indolent, not prone to the complications of tertiary syphilis, and subsides without leaving any trace. It may appear from the third month to the third year after infection. At a later time it appears as a tertiary syphiloma, rarely independent of sarcocele. An important consideration is that syphilitic epididymitis does not seem to destroy the permeability of the canal. The pathological anatomy has not been demonstrated, but is probably identical with that of other lesions of the same period. Syphilitic lesions of the spermatic ducts in other situations are underscribed, but they may nevertheless exist, as their painlessness and location would readily permit them to escape notice. It may follow from the foregoing train of reasoning that even if the semen be not infectious in itself, as has been demonstrated, it may acquire virulence in traversing a syphilitic epididymis, a urethra affected with mucous patches or other lesion possibly non-syphilitic in itself, but which allows a mixture of blood to take place during ejaculation. Such facts have an obvious bearing upon the doctrine of "syphilis by conception."—*Medical Bulletin.*

